# Age-related changes of human serum Sirtuin6 in adults

**DOI:** 10.1186/s12877-021-02399-0

**Published:** 2021-08-04

**Authors:** Ying Zhao, Xiangli Bai, Xiong Jia, Yajing Lu, Wenzhuo Cheng, Meng Shu, Yan Zhu, Lin Zhu, Li Wang, Yan Shu, Yi Song, Si Jin

**Affiliations:** Department of Endocrinology, Institute of geriatric medicine, Liyuan Hospital, Tongji Medical College, Huazhong University of Science and Technology, 39 Lake Road, East Lake Ecological Scenic, Wuhan, 430077 Hubei Province China

**Keywords:** Sirt6, Telomerase, Aging, Biomarker

## Abstract

**Background:**

Aging is a natural life process and with an aging population, age-related diseases (e.g. type 2 diabetes mellitus (T2DM), atherosclerosis-based cardiovascular diseases) are the primary mortality cause in older adults. Telomerase is often used as an aging biomarker. Detection and characterization of novel biomarkers can help in a more specific and sensitive identification of a person’s aging status. Also, this could help in age-related diseases early prevent, ultimately prolonging the population’s life span. Sirtuin 6 (Sirt6) - a member of the Sirtuins NAD^+^-dependent histone deacetylases family - is mainly intracellularly expressed, and is reported to be involved in the regulation of aging and aging-related diseases. Whether serum Sirt6 is correlated with aging and could be used as an aging biomarker is unknown. In the present study, we aimed to investigate the age-related Sirt6 changes in the serum of human adults.

**Methods:**

Participants were divided into three groups according to age: 20–30 years (Young); 45–55 years (Middle-aged); and ≥ 70 years (Old). The Sirt6 and telomerase serum concentrations were determined by ELISA. The Sirt6 and human telomerase reverse transcriptase (hTERT) expression in vessels from amputated human lower limbs were analyzed using real-time quantitative PCR (RT-qPCR) and immunohistochemical staining. The relationships between variables were evaluated by Pearson correlation analysis.

**Results:**

The Sirt6 and telomerase serum levels reduced with an increase in age. A similar tendency was observed for Sirt6 and hTERT in the vessel. Serum levels of Sirt6 were higher in females compared with males. Pearson’s regression analysis revealed that the Sirt6 serum level positively correlated with telomerase (*r* = 0.5743) and both were significantly negatively correlated with age (*r* = − 0.5830 and *r* = − 0.5993, respectively).

**Conclusions:**

We reported a negative correlation between serum Sirt6 concentration and aging in human beings. Therefore, the Sirt6 serum level is a potential sex-specific aging marker.

## Background

Aging - an irreversible process that is usually ignored - is characterized by a functional decline in cells, tissues, and organs. Currently, as life expectancy increases, the number of aging populations grows, leading to a remarkable increase in age-related chronic disease prevalence. To date, there is no practical method to predict aging and prevent aging-associated conditions.

Commonly, different aging markers are used, and none of them is purely specific to the human body’s aging [1]. Furthermore, translation of these biomarkers from bench side to bedside is a lengthy and costly process. Accurately and timely recognition of aging, precise estimation of its hazards, and effective intervention and prevention treatments have a great value to decrease aging-related diseases. Therefore, the development of more clinically promising early aging biomarkers is urgent.

The Sirtuins family has multiple enzymatic activities, including NAD^+^-dependent deacetylases and ADP-ribosyl transferases [2]. A member of this family, Sirtuin 6 (Sirt6), is involved in many aging and aging-related diseases regulatory pathways [3–5]. Previous studies reported that Sirt6 knockout mice displayed an aging-like phenotype [6], while rodent models with Sirt6 overexpression showed longevity phenotypes [7, 8]. Recently, it was identified that Sirt6 serves as an anti-aging protein. Therefore, we speculated that serum Sirt6 could be a potential aging biomarker.

The human telomerase is a ribonucleoprotein complex, composed of the reverse transcriptase catalytic subunit (TERT) and a telomerase RNA component (TERC) [9]. The hTERT expression is closely related to telomerase activity and serves as a limiting factor for telomerase activation [10]. Aging is highly associated with the development of telomere disorders and genomic instability [11–13]. Telomeres depletion and short telomeres accumulation occur in parallel with the human aging processes and maybe the underlying cause of aging-related diseases [14]. Meanwhile, the effect of Sirt6 on DNA repair and genomic integrity maintenance has been at the forefront of aging and aging-related disease research [15–17]. However, no study has yet explored the relationship between serum Sirt6 and human telomerase.

Therefore, we systematically analyzed age-related serum Sirt6 changes in healthy individuals and studied the correlation between serum Sirt6 and telomerase. We provided evidence that the Sirt6 age-related divergent expression pattern can be used as a clinical aging biomarker.

## Materials and methods

### Ethics statement

This study was approved by the Ethics Committee of Liyuan Hospital, Tongji Medical College, Huazhong University of Science and Technology. Written informed consents were obtained from all subjects and we performed this study according to the ethical principles for medical research involving human subjects detailed in the Declaration of Helsinki.

### Participants

A total of 155 individuals were recruited from the Liyuan Hospital of Tongji Medical College, Huazhong University of Science and Technology from September 2020 to November 2020 and divided by age into three groups: Young (24 males and 28 females, 20 to 30 years, 25.4 ± 0.4 years), Middle-aged (25 males and 36 females, 45 to 55 years, 50.1 ± 0.4 years), and Old (26 males and 16 females, ≥70 years, 74.9 ± 0.5 years). The inclusion criteria were: (1) fit the age requirements of each group; (2) healthy individuals; (3) willing to participate. The exclusion criteria were: (1) complicated with clinical disorders; (2) usage history of any medication within the past 3 months; (3) unwillingness to cooperate; (4) women in gestational and menstrual periods. All participants underwent a systemic physiological examination at the hospital, and their physiological functions were within the normal range.

### Human serum collection

After the provision of informed consent, participants were subjected to fasting conditions for at least 12 h. Blood samples were drawn from the cubital vein of subjects into 10 mL sterile-drying tubes. Then, samples were allowed to clot for 2 h at room temperature before centrifugation for 20 min at 1000×*g*. Freshly obtained serum samples were immediately processed or stored in aliquots for later use at − 80 °C.

### Enzyme-linked immunosorbent assay (ELISA)

A total of 155 serum samples were tested in the ELISA assay. They were categorized into three groups according to the age of individuals: Young (24 males and 28 females), Middle-aged (25 males and 36 females), and Old (26 males and 16 females). The serum concentrations of Sirt6 (ELISA Genie, Ireland) and telomerase (ELK Biotechnology, China), and Sirt6 deacetylase activities (abcam, USA) were examined using ELISA kits according to the manufacturer’s instructions. After 3 h of incubation at 37 °C, the optical density of each well was determined using a microplate reader at 450 nm. For each assay, the serum sample was diluted in a range of 1:1–1:100 into sample diluents. Duplicate assays were performed for each sample.

### Real-time quantitative PCR (RT-qPCR)

Lower extremity arterial blood vessels were obtained from patients who underwent lower-limb amputation due to severe traffic accidents. Patients were divided into three groups based on age: Young (*n* = 3, 24.6 ± 0.7 years); Middle-aged (*n =* 3, 49.6 ± 1.1 years) and Old (*n* = 5, 74.4 ± 0.8 years). These tissues also used for immunohistochemistry staining. Vessel tissues total RNA was extracted using TRIzol according to the manufacturer’s instructions (Invitrogen, CA, USA). RT-qPCR was performed using the SYBR Premix Ex Taq kit (TaKaRa, Tokyo, Japan) and detected by the iCycler Real-Time PCR Detection System (Bio-Rad, Hercules, USA). GAPDH was used as an internal control. The primer sequences were:

Sirt6: Forward: 5′-TGTGGAAGAATGTGCCAAGTGT-3′.

Reverse: 5′-AGCGATGTACCCAGCGTGAT-3′.

hTERT: Forward: 5′- CTCCCATTTCATCAGCAAGTTT-3′.

Reverse: 5′- CTTGGCTTTCAGGATGGAGTAG-3′.

GAPDH: Forward: 5′-CAGGAGGCATTGCTGATGAT-3′.

Reverse: 5′- GAAGGCTGGGGCTCATTT-3′.

### Immunohistochemistry

Human vessels samples of the lower extremity artery were collected from patients undergoing a lower-limb amputation due to severe traffic accidents. They were also divided into three subgroups based on age: Young (*n* = 3; 24.6 ± 0.7 years), Middle-aged (*n =* 3; 49.6 ± 1.1 years) and the Old (*n* = 5; 74.4 ± 0.8 years). We extracted a small fraction of the blood vessel tissue for IHC experiments. The tissues were fixed with 4% paraformaldehyde before embedding in paraffin for serial sectioning (5 μm) and incubated overnight at 4 °C with primary antibodies against Sirt6 (1:50; Proteintech, China), H3K9ac (1:100; Cell Signaling Technology, Beverly, MA, USA), H3K56ac (1:100; PTM BIO Technology Co., Ltd., Hangzhou, China) and hTERT (1200; abcam, USA). Next, the tissue sections were incubated with species-appropriate horseradish peroxidase-conjugated secondary antibodies (Absin, China) and analyzed, photographed using a microscope (Olympus, Tokyo, Japan), and quantified using the ImageJ software.

### Statistical analysis

Data were analyzed and managed using GraphPad Prism 5 version (GraphPad, San Diego, CA, USA) or SPSS 26 (SPSS Inc., Chicago, IL, USA). Data are expressed as mean ± SEM or percentages (%). For statistical comparison among the three groups, a one-way ANOVA test (continuous data) or a χ^2^ test (categorical data) were performed, followed by the Bonferroni correction for multiple comparisons. Two-way ANOVA with Bonferroni correction was used for comparisons between multiple groups. Correlations between variables were determined using Pearson correlation analysis. Statistical significance was set as *p* < 0.05.

## Results

### Baseline characteristics of the included participants

Participants were divided into three groups as described above and the detailed baseline characteristics of each group are summarised in Table 1. Following statistical analysis, we found that there were significant differences among groups regarding age (*p* < 0.001). However, no differences were detected regarding sex, Body Mass Index (BMI), smoking and drinking, blood pressure, blood glucose, glycosylated hemoglobin (HbA1c), lipoprotein lipid levels, alanine aminotransferase (ALT), aspartate aminotransferase (AST), and uric acid (UA).

**Table 1:** Baseline characteristics of the included participants

Parameters	Young (*n* = 52)	Middle (*n* = 61)	Old (*n* = 42)	*p*-value
Age (years)	25.4 ± 0.4^**a,b**^	50.1 ± 0.4^**b**^	74.9 ± 0.5	< 0.001
Female (%)	28 (53.85)	36 (59.01)	16 (38.1)	0.128^c^
Weight (kg)	61.5 ± 8.8	66.1 ± 12.1	64.7 ± 10.2	0.342^d^
BMI (kg/m2)	22.7 ± 3.2	23.3 ± 3.0	22.4 ± 2.5	0.441^d^
Smoking (%)	8 (15.38)	18 (29.51)	10 (23.81)	0.369^c^
Drinking (%)	12 (23.08)	12 (19.67)	12 (19.05)	0.157^c^
SBP (mm Hg)	116.25 ± 4.39	125.36 ± 5.26	128.46 ± 5.38	0.171^d^
DBP (mm Hg)	76.47 ± 1.42	78.25 ± 2.24	79.26 ± 3.14	0.203^d^
LDL-C (mmol/L)	2.0 ± 0.6	2.5 ± 0.6	2.8 ± 0.9	0.561^d^
HDL-C (mmol/L)	1.3 ± 0.2	1.2 ± 0.2	1.3 ± 0.3	0.457^d^
TG (mmol/L)	0.9 ± 0.2	1.0 ± 0.3	1.5 ± 0.9	0.614^d^
TC (mmol/L)	3.8 ± 0.8	4.3 ± 0.5	4.9 ± 1.2	0.648^d^
GFR (ml/min)	114.2 ± 13.5	109.7 ± 16.9	121.3 ± 25.9	0.798^d^
Cr (μmol/L)	73.9 ± 15.6	71.2 ± 12.3	81.7 ± 24.9	0.389^d^
AST (U/L)	18.6 ± 3.9	21.6 ± 6.2	25.4 ± 12.6	0.505^d^
ALT (U/L)	15.3 ± 7.2	24.1 ± 13.9	21.9 ± 16.7	0.661^d^
UA (μmol/L)	334.7 ± 58.2	310.1 ± 57.4	316.2 ± 61.3	0.298^d^
Glucose (mmol/L)	5.4 ± 0.2	5.3 ± 0.3	5.6 ± 0.7	0.412^d^
HbA1c (%)	4.6 ± 0.8	5.1 ± 0.7	4.8 ± 1.0	0.257^d^

Demographics and clinical characteristics of subjects

*Abbreviations*: *BMI* Body Mass Index, *SBP* systolic blood pressure, *DBP* diastolic blood pressure, *LDL-C* low-density lipoprotein cholesterol, *HDL-C* high-density lipoprotein cholesterol, *TG* triglyceride, *TC* total cholesterol, *GFR* glomerular filtration rate, *Cr* serum creatinine, *AST* aspartate transaminase, *ALT* alanine aminotransferase, *UA* uric acid, *HbA1c* glycosylated hemoglobin

Data are shown as mean ± SEM or percentage (%) of subjects in each group. ^a^ Significantly different vs Middle; ^b^ Significantly different vs Old. Statistical significance using ^c^ χ^2^ test for categorical data and ^d^one-way ANOVA test for continuous data followed by Bonferroni correction

### The serum levels of Sirt6 and telomerase gradually decreased with increasing age

**Fig. 1:**
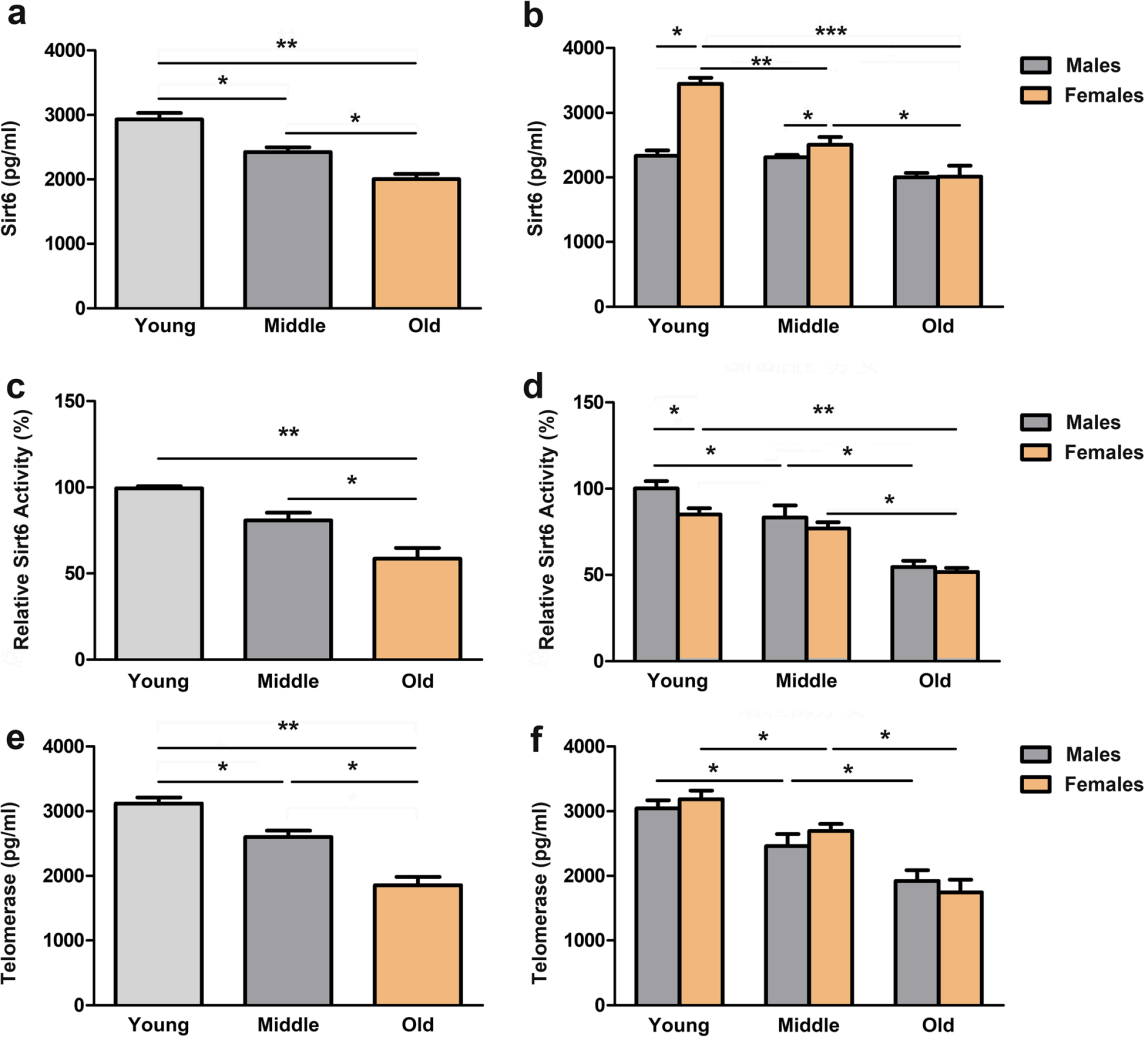
The serum levels of Sirt6 and telomerase in individuals of different ages. Sirt6 serum level was detected by ELISA in age groups (a) and male or female groups (b). The Sirt6 serum activity was detected by ELISA in age groups (c) and male or female groups (d). Telomerase serum level was detected by ELISA in age groups (e) and male or female groups (f). *p < 0.05, **p < 0.01, *** *p* < 0.001, one-way ANOVA and two-way ANOVA were used to compare significant differences followed by Bonferroni test

To understand the relationship between Sirt6 and age, we first measured serum Sirt6 levels in age groups using ELISA. We observed different serum Sirt6 levels among the Young, Middle-aged, and Old groups (Fig. 1a). Serum Sirt6 levels in middle-aged and elderly adults decreased significantly when compared to young adults. Further, we explored the relationship between sex and serum Sirt6 levels in different groups. The results indicated that females exhibited higher Sirt6 levels when compared to males in young and middle-aged groups. However, there was no significant difference regarding male subjects in different age groups (Fig. 1b). Also, we examined the Sirt6 deacetylase activity in each age group. With an increase in age, serum Sirt6 deacetylase activity in male subjects decreased gradually. Meanwhile, the serum Sirt6 deacetylase activity in the elderly group decreased in female subjects, while there was no significant difference between middle-aged and young groups (Fig. 1c-d). Currently, it has been well established that telomerase is considered an aging molecular characteristic [18]. Therefore, we measured the serum telomerase level in the three groups. Our analysis indicated that the telomerase levels decreased with increasing age, although no differences were observed between males and females (Fig. 1e-f). Altogether, we revealed that the changes in serum Sirt6 levels were related to aging, with sex differences.

### Pearson’s correlation analysis of Sirt6 levels based on age

**Fig. 2:**
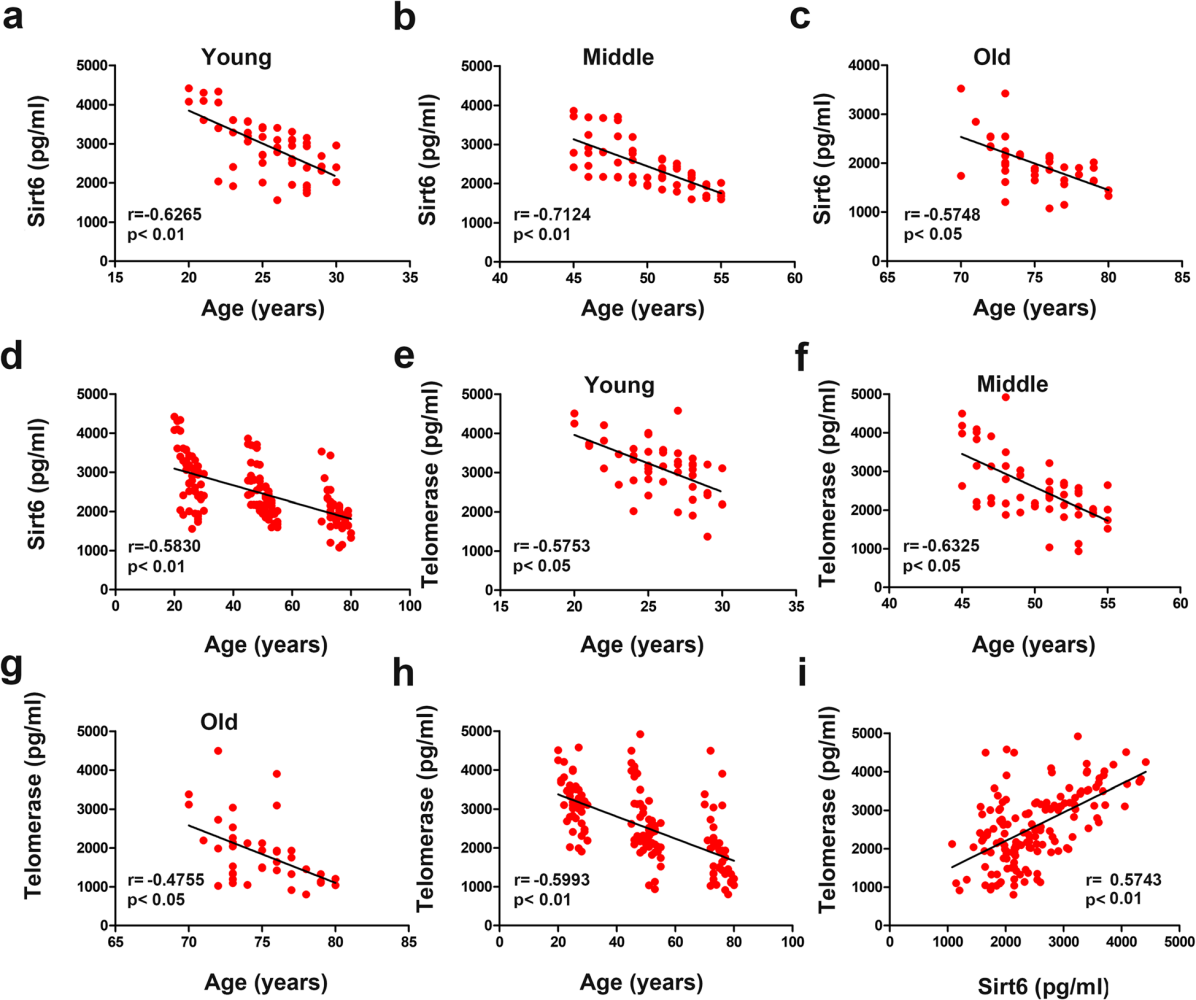
Sirt6 and telomerase relationship with age. (a-d) The linear relationship of Sirt6 with age-related changes in different age groups. (e-h) The linear relationship of telomerase with age-related changes in different age groups. (i) The linear relationship between Sirt6 and telomerase in healthy subjects. Correlations between variables were determined using Pearson correlation analysis. *p* < 0.05 was the significant level, and *r* represents Pearson’s correlation coefficients

To further verify the results mentioned above, we performed Pearson’s correlation analysis of Sirt6 levels based on age (Fig. 2a-d). Sirt6 expression was significantly inversely and linearly associated with age (*r* = − 0.5830). Additionally, we explored the relationship between telomerase and age (Fig. 2e-h). This analysis suggested that telomerase was also negatively correlated with age (*r* = − 0.5993). Therefore, we speculated that a linear relationship existed between Sirt6 and telomerase levels. Consistent with this hypothesis, the data revealed a stable and positive linear correlation between Sirt6 and telomerase (*r* = 0.5743) (Fig. 2i). These results demonstrated an overall significant and negative linear relationship between Sirt6 and age.

### Pearson’s correlation analysis of Sirt6 levels based on sex

**Fig. 3:**
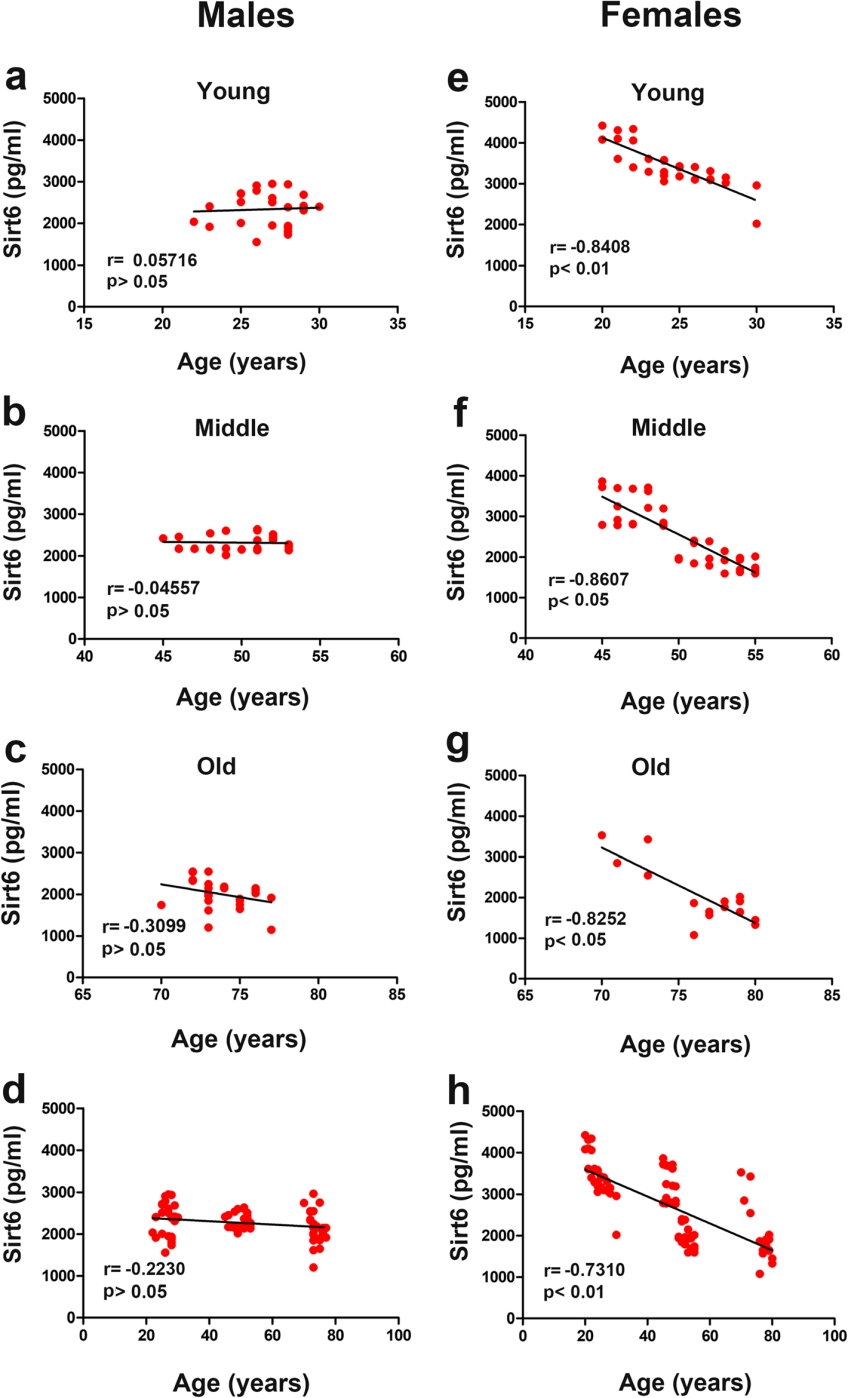
Regression analysis of Sirt6 levels based on sex. (a-d) The liner relationship of Sirt6 with age-related changes in males. (e-h) Sirt6 liner relationship with age-related changes in females. Correlations between Sirt6 levels and ages were determined using Pearson correlation analysis. *p* < 0.05 was the significant level, and *r* represents Pearson’s correlation coefficients

Considering the presence of differences regarding sex in serum Sirt6 levels, the regression analyses for males and females were performed separately. Pearson’s correlation analysis showed that there was no clear correlation between Sirt6 levels and different age groups for males (*r* = − 0.223) (Fig. 3a-d), while there was a highly significant correlation for females (*r* = − 0.731) (Fig. 3e-h). These results revealed that the differences of female Sirt6 component in young, middle aged, and elderly adults are responsible for the Sirt6 serum levels gradually decrease with age.

### The expression of Sirt6 in human vascular tissues decreases in an age-dependent manner

**Fig. 4:**
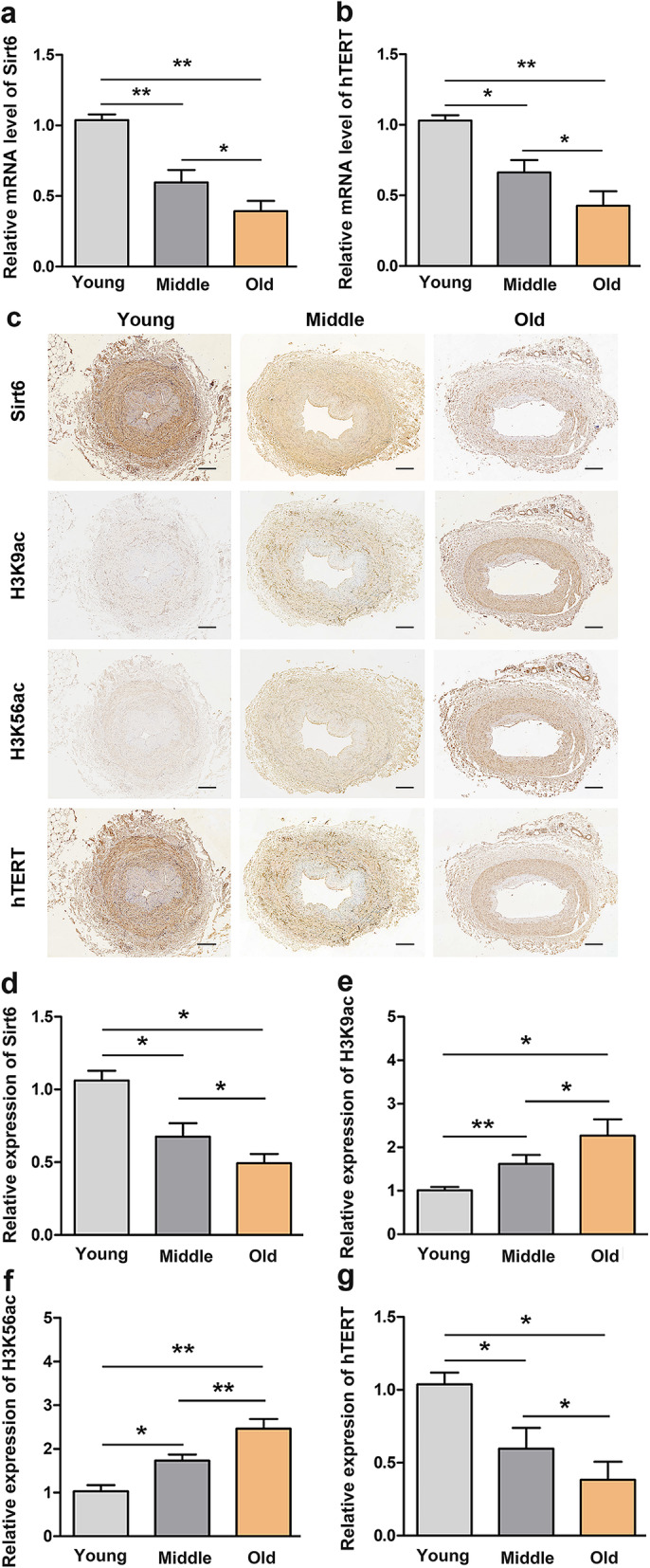
The expression of Sirt6 in vessels decreases in an age-dependent manner. Sirt6 (a) and hTERT (b) mRNA levels were measured by RT-qPCR in vascular tissue of different age groups. (c) Immunohistochemical staining of Sirt6, H3K9ac, H3K56ac, and hTERT in vascular tissues. (d-g) Representative immunohistochemical quantitative analysis of Sirt6/H3K9ac/H3K56ac/ hTERT-positive status in human vessels. Scale bars: 150 μm. * *p* < 0.05, ** *p* < 0.01, one-way ANOVA for multiple comparisons with Bonferroni test

Aging has been recognized as the largest risk factor for cardiovascular disease since vascular senescence is one of its major characteristics. Further, to verify the relationship between Sirt6 expression and aging, we collected lower extremity artery of young, middle and the elderly groups who were amputated due to serious traffic accidents. We analyzed Sirt6 expression and telomerase activity in vascular tissues in the three age groups. hTERT - an essential catalytic subunit to maintain telomerase activity - is consistent with telomerase activity [19]. We found that Sirt6 and hTERT mRNA levels markedly decreased in the elderly compared to young and middle-aged individuals (Fig. 4a-b). Similarly, the immunohistochemistry analysis showed that Sirt6 and hTERT expression gradually declined in an age-dependent manner and were lower in the Old group (Fig. 4c-g). Histone acetylation is a common post-translational modification that is closely related to cell senescence. It is well known that histone H3 lysine-9 (H3K9) and histone H3 lysinr-56 (H3K56) are Sirt6 deacetylase substrates. We observed that H3K9 and H3K56 acetylation was enhanced with increasing age in blood vascular tissues, which confirmed that Sirt6 expression was closely related to age. Overall, these results suggested that Sirt6 and hTERT levels were down-regulated with increasing age and might play a role in vascular senescence. Therefore, these findings provided clearly evidence that Sirt6 could be a potential biomarker for aging.

## Discussion

We demonstrated a negative correlation between serum deacetylase Sirt6 and aging, and that it can serve as a bonafide aging biomarker. These data can provide a basis for the development of novel prevention and intervention approaches against aging and age-related disorders.

The Sirtuin family of NAD^+^-dependent deacetylases plays an essential role in response to metabolic disorders, including T2DM. Previous studies have proven that Sirtuins - mammalian Sir2 - regulate aging/longevity in different model organisms [20, 21]. Among Sirtuins, Sirt6 acts as a longevity gene and exerts its beneficial effects regulating DNA damage, telomere maintenance and senescence. Many studies have reported that Sirt6 is an NAD^+^-dependent H3K9ac and H3K56ac deacetylase, protecting telomeres from damage through chromatin deacetylation [3, 22, 23]. First, our results demonstrated that Sirt6 was inversely related to increasing ages. Interestingly, we observed a difference in Sirt6 serum levels among young, middle-aged and old females, while there was no significant difference among males for each age group. This indicates that a decrease of Sirt6 in serum with increasing age is mainly related to serum Sirt6 component differences in females, suggesting that Sirt6 in serum plays a critical role in longevity and healthy aging in this sex. We mainly focused on Sirt6 deacetylase function, which protects against aging and different diseases through deacetylation and acetylation activity balancing. Also, we identified a significant difference in Sirt6 deacetylase activity among different age groups in males (Fig. 1d). We concluded that there is a decrease in Sirt6 deacetylase activity with increasing age in males. However, Sirt6 content did not change with age in males, indicating that the changes in Sirt6 deacetylase activity were predominant during the aging process of healthy males. Since Sirt6 is an anti-aging characteristic, our findings might help explain a worldwide observed phenomenon: women have a longer life span than men.

Telomeres are regions of repetitive DNA at the ends of chromosomes. They protect chromosomes from end-to-end fusions and DNA rearrangements [24]. Many clinical studies have reported that the telomere length shortening and telomerase activity reduction were related to aging and aging-associated diseases [25, 26]. The telomere length is negatively correlated with age [27], while there is no significant difference between males and females [28, 29]. Moreover, telomere extension by telomerase activity is generally considered the primary pathway to keep the telomere length [30]. Our study revealed that telomerase gradually decreased as age increased, and sex-specific differences were not observed. Sirt6 is associated with telomeres, and its depletion leads to telomere dysfunction and premature cellular senescence [31]. We measured Sirt6 and telomerase levels in different age groups, and a Pearson’s correlation analysis showed that increasing age strongly correlated with Sirt6 and telomerase decreases. Furthermore, an in-depth analysis confirmed that Sirt6 levels were markedly associated with telomerase. These results provide new evidence that combining detection of Sirt6 levels with telomerase levels could facilitate to sex-specific aging recognition in the future.

Vascular aging is closely associated with aging-related cardiovascular and cerebrovascular diseases and has garnered attention in current vascular disease research. There have been many advancements in understanding and elucidating the role of Sirtuins in senescent cells [32, 33]. However, the effect of Sirt6 on vascular aging remains unclear. Therefore, we attempted to explore Sirt6 role in vascular senescence. Our study showed that Sirt6 expression was remarkably lower in vessels of aged individuals. hTERT is the rate-limiting component of telomerase activity, essential for telomere length maintenance and anti-aging effects. Additionally, we observed that hTERT expression was associated with age and lower in aged individuals’ vessels. These results were consistent with previous reports stating that telomeres were progressively shortened with increased age in a substantial proportion of human cells [34, 35]. Also, the expression levels of H3K9ac and H3K56ac - known as Sirt6 substrates - were elevated in aging vessels, suggesting that the elderly population possesses higher levels of histone acetylation compared to young adults.

We found that Sirt6 levels in women were significantly higher than men in the young and middle-aged groups. The reason for the differences between sex may be related to genuine biological differences and hormonal variations. McCartney et al. reported that enrichment among differentially expressed genes in the testis may be indicative of a relationship with endocrine function and sex-specific aging [36]. Additionally, energy metabolism-related factors may also play an essential role in this sex difference. For instance, Nicotinamide adenine dinucleotide (NAD^+^) - a critical component of the respiratory chain on metabolism - serves as Sirtuins cofactor, including Sirt6. Indeed, NAD^+^ bioavailability exhibited distinct differences between males and females [37], and the cellular bioavailability of NAD^+^ declines upon aging [38].

The purpose of our research was to clarify the relationship between serum Sirt6 and age and determine whether serum Sirt6 can be used as an aging biomarker. For this, participants were divided into three subgroups based on age. As the transition period among young, middle-aged and elderly can be difficult and fuzzy, and many transition-aged people face multiple uncertain factors, the biological, psychological age, and social ages are inconsistent. To ensure that the age was the only variable, a 15 years gap between each group was necessary.

Although aging is a primary risk factor for the development of age-related diseases [39], researchers, unfortunately, often ignore the overall aging process when exploring the mechanisms of these diseases. Conversely, the effect of age-related diseases on aging acceleration is also vastly underestimated.

## Conclusions

We demonstrated that Sirt6 expression is associated with aging and can act as a specific aging biomarker, and compared with telomerase, Sirt6 is more gender-specific. Therefore, the combined detection of serum Sirt6 and telomerase will have higher accuracy in the aging identification for different genders. Since Sirt6 exerts beneficial effects on cellular anti-senescence, whether a supplement of serum Sirt6 could delay the aging process and prevent or treat aging-related diseases might be an interesting project to study in the future.

## Data Availability

The datasets generated and/or analysed during the current study are not available due research design but are available from the corresponding author on reasonable request.

## Abbreviations

Sirt6: Silent information regulator 6
T2DM: Type 2 diabetes mellitus
hTERT: Human telomerase reverse transcriptase
SEM: Standard error of the mean
